# Biochar and Quicklime Co-Application Enhances Soil Fertility and Nut Yield in Acidic Pecan Orchards

**DOI:** 10.3390/plants15101566

**Published:** 2026-05-20

**Authors:** Jiajun Li, Juan Xie, Longfei Wang, Junqin Zhou, Jun Yuan

**Affiliations:** 1 State Key Laboratory of Woody Oil Resources Utilization, Central South University of Forestry and Technology, Changsha 410004, China; 13787502398@163.com (J.L.); 19848180468@163.com (J.X.); 13233817158@163.com (L.W.); zhoujunqin@csuft.edu.cn (J.Z.); 2 Yuelu Mountain Laboratory, Central South University of Forestry and Technology, Changsha 410004, China; 3 College of Landscape Architecture, Central South University of Forestry and Technology, Changsha 410004, China

**Keywords:** *Carya illinoinensis*, red soil, nutrient availability, kernel quality, fruit quality

## Abstract

Soil acidification and low nutrient availability in acidic red soils are major constraints on pecan (*Carya illinoinensis*) productivity and fruit quality. However, the integrated effects of quicklime and biochar application in pecan orchards on acidic red soils remain poorly understood. In this context, a field experiment was conducted in an 8-year-old pecan orchard in an acidic red soil region to evaluate the effects of sole and combined applications of biochar and quicklime at different ratios on soil properties, fruit yield, and quality. The results showed that the combined application of biochar and quicklime showed greater benefits for soil fertility, fruit yield, and kernel quality than single-amendment treatments. The combined treatments significantly increased soil pH by 0.47–2.15 units relative to the control and markedly improved soil nutrient status. After 12 months of application, SOM contents under L1B–L3B were 20.8–23.2% higher than those under the corresponding quicklime-only treatments, reaching 37.57–43.37 g·kg^−1^. The combined treatments also maintained higher total nitrogen, total phosphorus, and available potassium levels than the corresponding quicklime-only treatments, with TN under L1B–L3B reaching 1.65–1.78 g·kg^−1^, representing a 126.0–166.7% increase over their respective quicklime-only treatments. The combined treatments also generally enhanced soil biological activity and improved fruit yield and quality. Their effects on fruit traits varied with application ratio: the low-dose treatment (L1B, 2 kg biochar + 1 kg quicklime) was more effective in improving physical traits such as dry kernel weight and kernel percentage; the medium-dose treatment (L2B, 2 kg biochar + 1.5 kg quicklime) produced the highest single-tree yield, reaching 26.80 kg·tree^−1^, which was 24.25% higher than the control and significantly higher than all single-amendment treatments (23.43–25.07 kg·tree^−1^); and the high-dose treatment (L3B, 2 kg biochar + 2 kg quicklime) was more favorable for improving nutritional quality, increasing amino acid and vitamin E contents to 1267.01 μg·kg^−1^ and 153.22 μg·g^−1^, respectively, which were 45.41–91.90% and 5.02–78.77% higher than those under the single-amendment treatments. Overall, the combined application of biochar and quicklime effectively alleviated soil acidification, improved soil fertility, and promoted higher fruit yield and quality, providing a scientific basis for the efficient, high-quality, and sustainable development of pecan orchards in acidic red soil regions.

## 1. Introduction

Pecan (*Carya illinoinensis*) is rich in unsaturated fatty acids, high-quality proteins, and various bioactive compounds. These attributes confer high nutritional value and considerable potential for processing and utilization [1,2]. With the development of the global woody oil crop industry and specialty nut industry, the cultivation area of pecan has gradually expanded in many regions of China. It has become one of the most promising economic tree species in the subtropical regions of southern China [3,4]. For such perennial fruit trees, sustained and stable yield formation and quality improvement depend not only on cultivar selection and cultivation management but also on the long-term stability of the rhizosphere soil environment. However, most pecan cultivation areas in southern China are located in subtropical hilly and mountainous regions. In these areas, acidic red soils are widely distributed. These soils generally suffer from high acidity, low nutrient availability, and high potential toxicity risks from aluminum, manganese, and other elements. Such conditions are unfavorable for the accumulation of soil organic matter and the maintenance of biological activity. Consequently, they constrain root growth, tree nutrient allocation, and fruit development [5,6,7,8,9]. In addition, high temperature, heavy rainfall, intense leaching, and long-term fertilization have further intensified orchard soil acidification [5,6]. Therefore, alleviating soil acidification and maintaining soil fertility while improving fruit yield and quality have become key issues for efficient and high-quality pecan production in red soil regions.

Currently, the application of alkaline amendments is an important measure for alleviating soil constraints in acidic orchards. Among these, quicklime is widely applied due to its strong alkalinity, low cost, and rapid efficacy. Quicklime can rapidly neutralize soil acidity, alleviate acid stress, and improve the rhizosphere environment in the short term [10,11]. However, its effects are mostly limited to rapid regulation with relatively limited persistence, and excessive application may also lead to nutrient imbalance, accelerated depletion of soil organic matter, and inhibition of certain biological processes [11,12]. In contrast, biochar, owing to its higher pH, abundant pore structure, and strong surface adsorption capacity, is regarded as a carbon-based material with potential for both soil amendment and fertility enhancement [12]. Previous studies have indicated that biochar can enhance the buffering capacity of acidic soils, promote organic matter accumulation and nutrient retention, and provide relatively stable habitats for soil microorganisms. However, its capacity for rapid acidity adjustment is generally weaker than that of quicklime, and its amelioration effects are readily influenced by factors such as feedstock type, pyrolysis conditions, application rate, and soil background [12]. Therefore, quicklime and biochar exhibit distinct advantages of “rapid deacidification” and “sustained fertility enhancement,” respectively, in the amelioration of acidic soils, and their combined application is thus considered to have potential synergistic effects [12,13,14,15].

Although previous studies have shown that the combined application of quicklime and biochar can increase soil pH, reduce exchangeable aluminum levels, improve nitrogen cycling processes, and promote crop growth, these findings are mainly derived from studies on general acidic soils and annual cropping systems such as maize and legumes [10,13,14,15]. For perennial woody fruit trees, especially the in situ responses and production effects under mature orchard conditions, systematic research remains lacking. In addition, existing studies have mainly focused on soil acidity or changes in single nutrients, while the coordinated responses of soil physicochemical properties, biological activities, and fruit yield and quality remain unclear [16,17].

Therefore, this study aimed to evaluate whether the co-application of biochar and quicklime could alleviate soil acidification, improve soil fertility and biological properties, and enhance nut yield and quality in acidic red soil pecan orchards. The study further examined changes in soil acidity, nutrient availability, enzyme activity, and microbial abundance, and explored their associations with pecan yield formation and quality improvement. These findings are expected to provide a scientific basis for sustainable soil management and high-quality pecan production in acidic red soil regions.

## 2. Results

### 2.1. Effects of Different Treatments on Soil Chemical Properties

**Figure 1:**
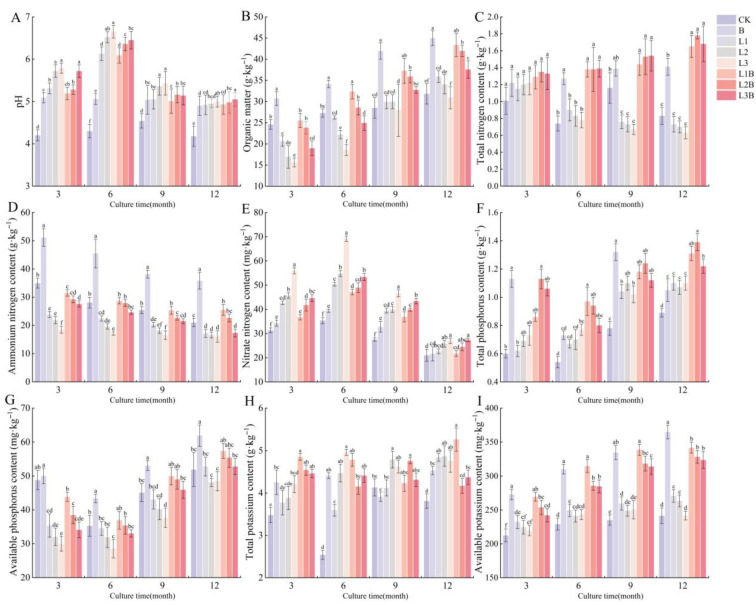
Effects of different treatments on soil chemical properties. (**A**) pH. (**B**) Soil organic matter (SOM) content (g·kg^−1^). (**C**) Total nitrogen (TN) content (g·kg^−1^). (**D**) Ammonium nitrogen (NH_4_^+^-N) content (g·kg^−1^). (**E**) Nitrate nitrogen (NO_3_^−^-N) content (mg·kg^−1^). (**F**) Total phosphorus (TP) content (g·kg^−1^). (**G**) Available phosphorus (AP) content (mg·kg^−1^). (**H**) Total potassium (TK) content (g·kg^−1^). (**I**) Available potassium (AK) content (mg·kg^−1^). Different letters denote significant differences between treatments at *p* < 0.05.

All treatments significantly increased soil pH throughout the experimental period (*p* < 0.05) (Figure 1A). In all quicklime-containing treatments, soil pH increased rapidly after application, peaked at 6 months, and then gradually declined, but remained significantly higher than that in the CK at 12 months; in contrast, the biochar-only treatment showed a more gradual change. Among these treatments, the higher-dose quicklime-only treatments (L2 and L3) produced the greatest increases in soil pH at the peak stage, with increases of 0.77–2.22 and 0.82–2.35 pH units relative to the CK, respectively (*p* < 0.05). The combined treatments (L1B–L3B) likewise significantly increased soil pH relative to the CK, with increases of 0.47–1.79, 0.62–2.06, and 0.60–2.15 units, respectively (*p* < 0.05). Soil pH in L2B and L3B was significantly lower than that in L2 and L3, respectively, at 3–9 months (*p* < 0.05), whereas no significant differences were detected between these combined treatments and their corresponding quicklime-only treatments at 12 months (*p* > 0.05). No significant differences were observed between L1B and L1 at any sampling time point (*p* > 0.05). At most time points, all combined treatments exhibited significantly higher soil pH than the biochar-only treatment (*p* < 0.05), and this pH-enhancing advantage became more pronounced with increasing quicklime application rates.

Across all treatments, SOM contents increased progressively over time and peaked at 12 months after application, although some treatments initially showed lower SOM contents than the CK before gradually recovering (Figure 1B). In all combined treatments, SOM content was significantly higher than that in the corresponding quicklime-only treatments (*p* < 0.05). At 12 months, SOM contents in L1B, L2B, and L3B reached 43.37, 41.96, and 37.57 g·kg^−1^, respectively, which were 20.8%, 23.2%, and 21.6% higher than those in the corresponding quicklime-only treatments L1, L2, and L3, respectively. However, within the combined treatments, SOM content declined with increasing quicklime application rate. Although the biochar-only treatment maintained the highest SOM content throughout the sampling periods, no significant difference was observed between L1B and B from month 6 onward (*p* > 0.05), and both treatments were significantly higher than the CK (*p* < 0.05). In addition, the SOM content in L2B did not differ significantly from that in the CK at 3 and 6 months after application (*p* > 0.05). By 12 months, all combined treatments showed significantly higher SOM contents than the CK (*p* < 0.05), with increases of 36.31%, 31.86%, and 18.08%, respectively.

Overall, nutrient responses indicated that combined applications exerted a more stable enhancing effect than quicklime-only application, although the responses varied among nutrient forms. For nitrogen (Figure 1C–E), TN contents were highest in the combined treatments across all sampling periods. Differences among treatments were not significant at 3 months; however, from 6 months onward, all combined treatments were significantly higher than the CK and the corresponding quicklime-only treatments (*p* < 0.05) and maintained relatively high levels from 9 to 12 months. Specifically, at 12 months, the increases in TN for L1B, L2B, and L3B relative to their corresponding quicklime-only treatments reached 126.0%, 154.3%, and 166.7%, respectively (*p* < 0.05). The temporal dynamics of NO_3_^−^-N were generally consistent with those of TN, with relatively high levels usually observed at 6 months, and all combined treatments were significantly higher than the CK and the biochar-only treatment between 6 and 9 months, with more pronounced increases in L2B and L3B at 12 months. In contrast, NH_4_^+^-N generally declined over time. Quicklime-only treatments resulted in overall lower values, whereas the biochar-only treatment was significantly higher than the CK across all sampling periods (*p* < 0.05). The combined treatments showed only a limited sustaining effect in the later stages. For phosphorus (Figure 1F,G), the combined treatments were significantly higher than the CK from 6 months onward and maintained elevated TP contents from 9 to 12 months. At 12 months, TP in L2B and L1B reached 1.39 and 1.31 g·kg^−1^, respectively, both significantly higher than in the corresponding quicklime-only treatments (*p* < 0.05). In comparison, AP remained at relatively high levels under combined application, with L1B and L2B performing better in the mid-to-late stages, but overall remained lower than under the biochar-only treatment. For potassium (Figure 1H,I), L1B showed the strongest response, with TK reaching the highest levels at both 3 and 12 months. Meanwhile, AK in L1B reached 314.67 and 338.67 mg·kg^−1^ at 6 and 9 months, respectively. All combined treatments maintained relatively high AK levels and were significantly higher than their corresponding quicklime-only treatments across all sampling periods (*p* < 0.05). However, at 12 months, AK under the biochar-only treatment was the highest and significantly exceeded that in all combined treatments (*p* < 0.05).

### 2.2. Effects of Different Treatments on Soil Enzyme Activities

**Figure 2:**
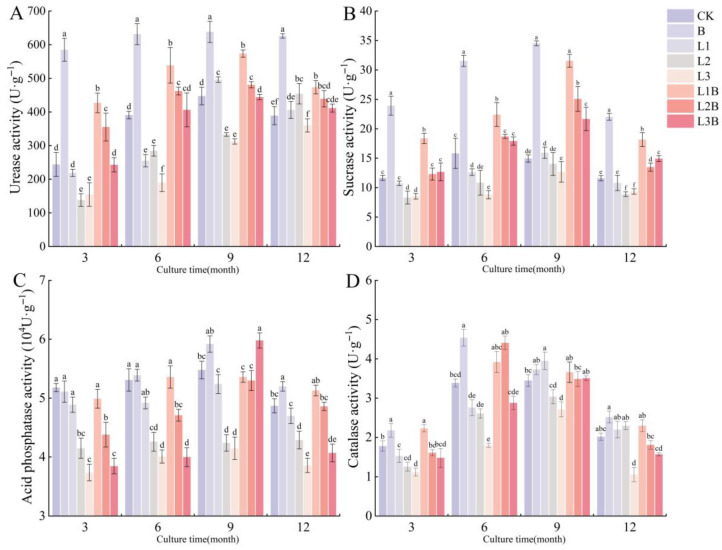
Effects of different treatments on soil enzyme activities. (**A**) Urease (UE) activity (U·g^−1^). (**B**) Sucrase (SC) activity (U·g^−1^). (**C**) Acid phosphatase (ACP) activity (104 U·g^−1^). (**D**) Catalase (CAT) activity (U·g^−1^). Different letters denote significant differences between treatments at *p* < 0.05.

The responses of UE and SC to different treatments were relatively consistent, with all combined treatments generally outperforming their corresponding quicklime-only treatments. Regarding UE (Figure 2A), L1B reached 538.40 and 573.60 U·g^−1^ at 6 and 9 months, respectively, both significantly higher than L1 (*p* < 0.05); L2B was also significantly higher than L2 at 3, 6, and 9 months (*p* < 0.05). In contrast, L3B showed weaker enhancement of UE, with no significant difference from CK in most periods (*p* > 0.05). For SC (Figure 2B), L1B was significantly higher than L1 across all four periods (*p* < 0.05), reaching 31.55 U·g^−1^ at 9 months, and showed no significant difference from the biochar-only treatment (*p* > 0.05); L2B was significantly higher than L2 at 6, 9, and 12 months (*p* < 0.05), and L3B was also higher than its corresponding quicklime-only treatment during the same periods. For both UE and SC, the biochar-only treatment maintained the highest levels across all sampling periods.

In contrast, CAT and ACP exhibited greater fluctuations in their responses to the treatments. CAT exhibited the advantages of combined application only in certain periods (Figure 2D); L1B reached 2.23 U·g^−1^ at 3 months, the highest value at that time, and was significantly higher than L1 and CK (*p* < 0.05); L2B increased to 4.41 U·g^−1^ at 6 months, significantly higher than L2 (*p* < 0.05), and showed no significant difference from the biochar-only treatment (*p* > 0.05). High-dose quicklime-only application resulted in overall lower levels, with L3 being the lowest among all treatments at 12 months (*p* < 0.05). ACP did not show stable advantages of combined application in the early stage, with L2B and L3B being significantly lower than CK at 3–6 months (*p* < 0.05); by 9 months, L3B increased to 5.98 × 10^4^ U·g^−1^, the highest value among all treatments, and was significantly higher than CK (*p* < 0.05), before declining again at 12 months (Figure 2C).

### 2.3. Effects of Different Treatments on Soil Microbial Abundance

**Figure 3:**
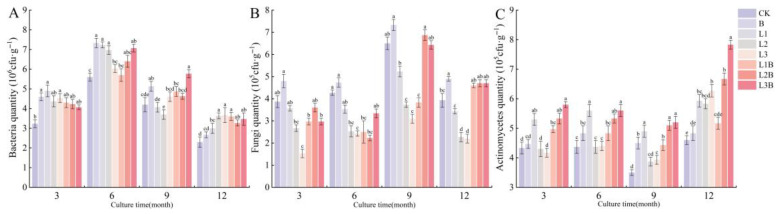
Effects of different treatments on soil microorganisms. (**A**) Bacteria (TPC) quantity (10^6^ cfu·g^−1^). (**B**) Fungi (TFC) quantity (10^5^ cfu·g^−1^). (**C**) Actinomycetes (TAC) quantity (10^5^ cfu·g^−1^). Different letters denote significant differences between treatments at *p* < 0.05.

Regarding bacterial and actinomycete abundances, the combined treatments exhibited more pronounced advantages in the later stages. For bacterial abundance (Figure 3A), only L3B was significantly higher than CK at 9 months (*p* < 0.05), while the remaining combined treatments showed no significant difference from CK (*p* > 0.05); by 12 months, L1B, L2B, and L3B had increased to 3.60, 3.27, and 3.47 × 10^6^ cfu·g^−1^, respectively, all significantly higher than both the biochar-only treatment and CK (*p* < 0.05), yet not significantly different from L2 and L3 (*p* > 0.05). Actinomycete abundance responded more markedly to the combined treatments (Figure 3C), with L3B reaching 5.80, 5.60, 5.20, and 7.83 × 10^5^ cfu·g^−1^ at 3, 6, 9, and 12 months, respectively; these values were the highest in each period and significantly higher than CK (*p* < 0.05). In particular, L3B at 12 months was also significantly higher than all other treatments (*p* < 0.05).

In contrast, fungal abundance exhibited a less stable response to the combined application (Figure 3B). At 12 months, fungal abundance in the combined treatments ranged from 4.60–4.70 × 10^5^ cfu·g^−1^, showing no significant difference from CK (*p* > 0.05) but being significantly higher than L2 and L3 (*p* < 0.05). At 9 months, L2B increased to 6.87 × 10^5^ cfu·g^−1^, showing no significant difference from the biochar-only treatment (*p* > 0.05), whereas L1B was significantly lower than CK at both 6 and 9 months (*p* < 0.05).

### 2.4. Effects of Different Treatments on Fruit Yield and Economic Traits

**Figure 4:**
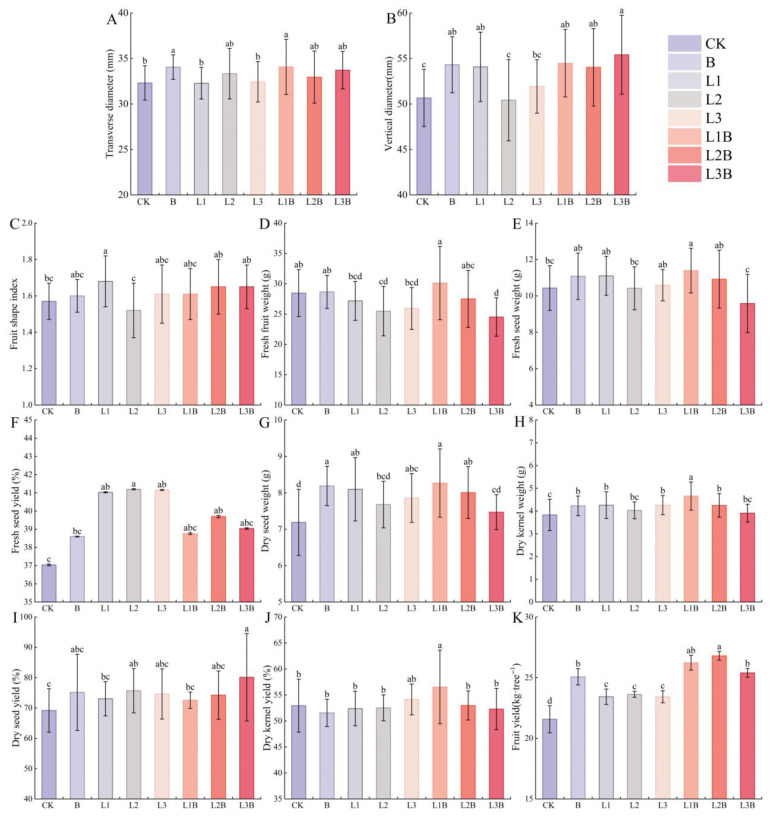
Effects of different treatments on fruit yield and economic characteristics. (**A**) Transverse diameter (FTD) (mm). (**B**) Vertical diameter (FVD) (mm). (**C**) Fruit shape index (FSI). (**D**) Fresh fruit weight (FFY) (g). (**E**) Fresh seed weight (FSW) (g). (**F**) Fresh seed yield (FSY) (%). (**G**) Dry seed weight (DSW) (g). (**H**) Dry kernel weight (DKW) (g). (**I**) Dry seed yield (DSY) (%). (**J**) Dry kernel yield (DKY) (%). (**K**) Fruit yield (FY) (kg·tree^−1^). Different letters denote significant differences between treatments at *p* < 0.05.

Overall, treatment effects on fruit morphological traits were relatively limited, whereas clearer differences were observed for several fresh-fruit and dried-fruit traits and for yield (Figure 4A–K). FTD was greatest under L1B (34.07 mm), showing no significant difference from the sole application of biochar (34.05 mm), but remaining significantly higher than that under CK and L1 (*p* < 0.05). FVD was highest under L3B (55.41 mm), significantly exceeding that under CK and L3 (*p* < 0.05). FSI varied only slightly among the combined treatments (1.61–1.65), while the highest value among the sole applications was recorded in L1 (1.68).

Among the fresh-fruit traits, FFY was highest in L1B (30.11 g), significantly higher than in all sole applications of quicklime (*p* < 0.05), but not significantly different from the sole application of biochar or CK (*p* > 0.05). FSW also reached its maximum in L1B (11.39 g), significantly higher than that under CK and L2 (*p* < 0.05), but comparable to that under the other sole applications (*p* > 0.05). FSY ranged from 38.76% to 39.70% across the combined treatments, with no significant differences relative to the sole applications (*p* > 0.05).

Among the dried-fruit traits, DSW was highest in L1B (8.27 g), significantly higher than that under CK (*p* < 0.05), but not significantly different from that under the sole application of biochar or L1 (*p* > 0.05). DKW and DKY were likewise highest in L1B, at 4.66 g and 56.54%, respectively. In particular, DKW in L1B was significantly higher than in all other treatments (*p* < 0.05), and DKY was significantly higher than that under B and L1 (*p* < 0.05). DSY reached its maximum under L3B (80.13%), significantly higher than that under CK (*p* < 0.05). Yield was highest in L2B among the combined treatments (26.80 kg·tree^−1^), significantly exceeding that of all sole applications (*p* < 0.05).

### 2.5. Effects of Different Treatments on the Major Nutritional Components of Fruits

**Figure 5:**
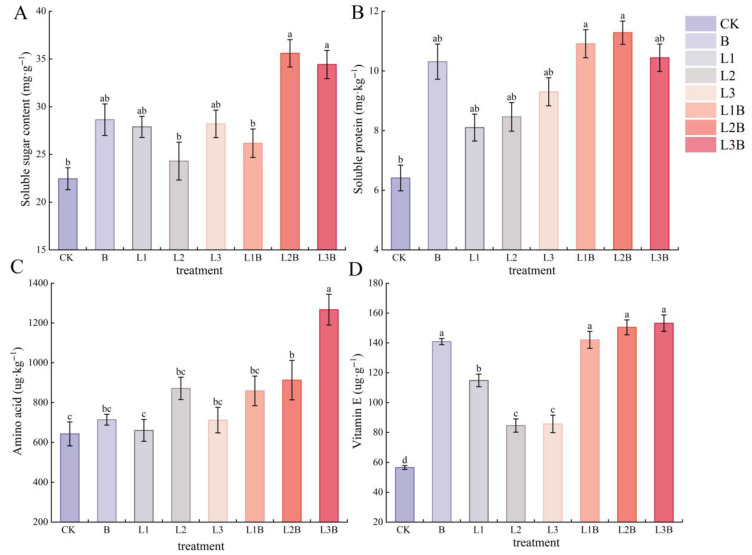
Effects of different treatments on the major nutritional components of fruits. (**A**) Soluble sugar (SS) content (mg·g^−1^). (**B**) Soluble protein (SP) (mg·kg^−1^). (**C**) Amino acid (AA) (μg·kg^−1^). (**D**) Vitamin E (VE) (μg·g^−1^). Different letters denote significant differences between treatments at *p* < 0.05.

Among the nutritional component indices, SS was higher under L2B and L3B (35.59 and 34.42 mg·g^−1^, respectively), both significantly higher than CK and L2 (*p* < 0.05), with percentage increases of 58.53% and 53.32% compared to CK (Figure 5A). SP was relatively high under L1B and L2B, reaching 10.91 and 11.28 mg·kg^−1^, respectively. Although these values were numerically higher than those under all single-amendment treatments, the differences were not significant (*p* > 0.05). Both L1B and L2B were significantly higher than CK (*p* < 0.05), with increases of 70.20% and 75.98%, respectively. In addition, although L2B exhibited the numerically highest SP content, no significant differences were detected among the combined treatments (*p* > 0.05), indicating that increasing the quicklime rate beyond L1B did not provide a statistically significant additional benefit for soluble protein accumulation. In contrast, AA and VE exhibited more pronounced responses to the treatments (Figure 5C,D). AA was highest under L3B (1267.01 μg·kg^−1^), significantly higher than all other treatments (*p* < 0.05), increasing by 97.09%, 77.48%, and 77.97% compared to CK, B, and L3, respectively. Vitamin E content was relatively high in all combined treatments and the B treatment, and all combined treatments were significantly higher than CK and all sole applications of quicklime (*p* < 0.05), with L3B being the highest at 153.22 μg·g^−1^, representing a 170.80% increase compared to CK (*p* < 0.05).

### 2.6. Effects of Different Treatments on the Oil Content and Fatty Acid Composition of Pecan Kernels

**Figure 6:**
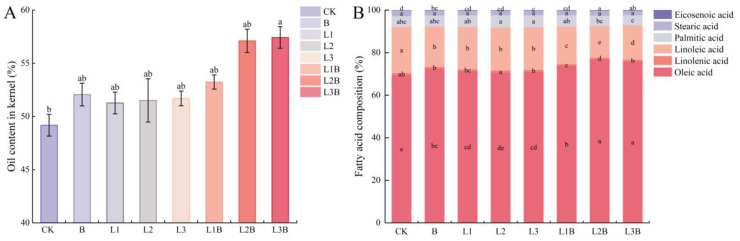
(**A**) Effects of biochar and quicklime application on oil content of Carya illinoensis. (**B**) Effects of biochar and quicklime application on fatty acid composition of Carya illinoensis. Different letters denote significant differences between treatments at *p* < 0.05.

In terms of oil content (Figure 6A), only the L3B treatment was significantly higher than CK (*p* < 0.05), with an oil content of 57.43% and an increase of 16.78%; the remaining treatments, although all showing an increasing trend, did not differ significantly from CK (*p* > 0.05). In terms of fatty acid composition (Figure 6B), all treatments generally exhibited an increase in oleic acid and a decrease in linoleic acid. Except for the L2 treatment, the oleic acid content in all other treatments was significantly higher than that under CK (*p* < 0.05). Meanwhile, the oleic acid content under all combined treatments was significantly higher than that under the corresponding sole application of quicklime. Among these, L2B and L3B were the most pronounced, reaching 76.98% and 75.81%, respectively, and both were significantly higher than the sole application of biochar. Correspondingly, the linoleic acid content under all fertilizer treatments was significantly lower than that under CK (*p* < 0.05), and all combined treatments were significantly lower than the corresponding sole application of quicklime and sole application of biochar, with L2B and L3B decreasing by 31.81% and 24.88%, respectively, compared to CK. The changes in the other fatty acid components were relatively small. Linolenic acid overall showed a downward trend, with only the L2 treatment exhibiting a slight increase. Palmitic acid did not differ significantly among the treatments (*p* > 0.05). Eicosenoic acid overall showed an increasing trend, with L2B and L3B exhibiting significantly greater increases than CK, at 0.14% and 0.12%, respectively (*p* < 0.05).

### 2.7. Correlation Analysis Between Soil Properties and Fruit Quality

**Figure 7:**
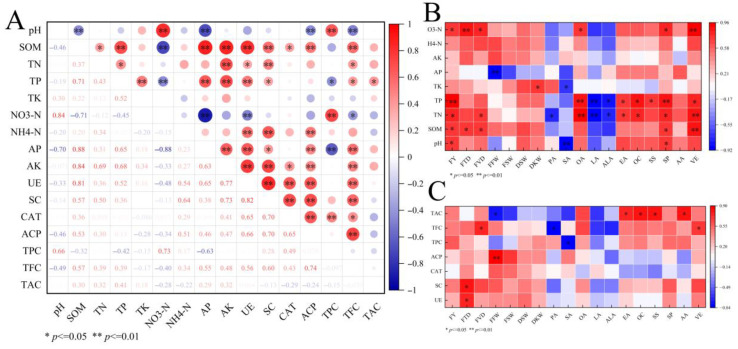
(**A**) Correlation analysis between soil nutrient content and enzyme activity. (**B**) Correlation analysis between soil nutrient content and fruit quality. (**C**) Correlation analysis between enzyme activity and fruit quality.

Pearson correlation analysis revealed strong associations among soil chemical properties, as well as between these properties and fruit traits, whereas correlations between soil biological indicators and fruit traits were relatively limited (Figure 7A–C). Among the soil chemical properties, pH was positively correlated with NO_3_^−^-N (*p* < 0.01) but negatively correlated with SOM and AP (*p* < 0.01). SOM was positively correlated with TN (*p* < 0.05) as well as with TP, AP, and AK (*p* < 0.01), but negatively correlated with NO_3_^−^-N (*p* < 0.01). TP was also positively correlated with TK, AP, and AK (*p* < 0.01).

Most soil enzyme activities were positively associated with SOM, NH_4_^+^-N, and several phosphorus- and potassium-related variables; moreover, all four enzyme activities exhibited significant positive pairwise correlations (*p* < 0.01). Microbial abundance showed differential responses to soil properties: TPC was positively correlated with pH and NO_3_^−^-N (*p* < 0.01), whereas TFC was negatively correlated with pH (*p* < 0.01) but positively correlated with SOM and several available nutrient variables (*p* < 0.01).

More significant correlations were observed between fruit traits and soil chemical properties. Fruit yield was positively correlated with pH, SOM, and NO_3_^−^-N (*p* < 0.05). NO_3_^−^-N was positively correlated with FTD (*p* < 0.01) and FVD (*p* < 0.05), while SOM was also positively correlated with both FTD and FVD (*p* < 0.05). Among the quality traits, TN and TP were positively correlated with oleic acid (*p* < 0.01) but negatively correlated with linoleic acid (*p* < 0.01), and were further positively correlated with oil content and eicosenoic acid (*p* < 0.05). SP and VE were mainly positively associated with pH, SOM, and nitrogen- and phosphorus-related nutrients, whereas AA showed no significant correlations with soil nutrient variables. In contrast, fruit yield and its major component traits generally exhibited no significant correlations with soil enzyme activities or microbial abundance, with significant associations limited to only a few individual morphological and quality traits.

## 3. Discussion

The present study aimed to evaluate the comprehensive effects of the combined application of biochar and quicklime on soil amelioration, yield formation, and fruit quality enhancement in pecan orchards established on acidic red soil. The results demonstrated that the combined application could simultaneously alleviate soil acidity, improve nutrient status, and enhance selected biological activities, thereby further promoting improvements in fruit economic traits and nutritional quality. Different combined treatments exhibited distinct dose-dependent differentiation with respect to yield and fruit quality: L1B proved more advantageous for improving fruit physical traits, L2B proved more advantageous for stabilizing and increasing yield, whereas L3B was more effective in enhancing nutritional quality and lipid composition. These findings suggest that the combined application of biochar and quicklime in pecan orchards serves not merely as a measure for acidity correction and nutrient optimization, but rather as a promising integrated amelioration strategy capable of concurrently achieving both yield and quality objectives [10,18,19].

### 3.1. Combined Application of Biochar and Quicklime Improves Soil Chemical Properties

Firstly, the changes in soil pH and SOM indicate that biochar and quicklime have complementary roles in the amelioration of acidic soils. Compared with the sole application of biochar, the combined application produced a clear additive benefit, and this benefit became more pronounced with increasing quicklime application rates. Previous reviews and meta-analyses have shown that biochar can alleviate soil acidification and enhance acid-buffering capacity, although the magnitude of this effect is jointly determined by biochar properties and soil background conditions [12,20,21]. Consistent with these findings, the combined application in the present study significantly increased soil pH and, at the later stage of the experiment, achieved an amelioration effect comparable to that of the sole application of quicklime at the corresponding rate. This suggests that quicklime primarily provides rapid neutralization in the early stage, whereas biochar contributes more to the long-term maintenance of buffering capacity, further supporting the synergistic pattern of rapid effects from lime and longer-lasting effects from biochar [13,22]. Meanwhile, the highest SOM values were consistently observed under the sole application of biochar, and both L1B and L2B remained at relatively high levels during the middle and later stages, whereas the increase in L3B was comparatively delayed. This indicates that a moderate lime input did not substantially weaken the carbon-retention effect of biochar, whereas a higher lime input may reduce the marginal contribution of biochar to carbon accumulation [13,23]. In addition, L3B exhibited a stage-dependent pattern, with SOM declining initially and then increasing again at the later stage. This pattern is consistent with the view that pH elevation can alleviate acid stress and stimulate decomposition processes, thereby accelerating organic carbon turnover [24,25]. Such temporary SOM loss under high lime input may also be associated with a positive priming effect. In this process, biochar-derived carbon, particularly in combination with a strong alkaline amendment, may transiently accelerate the mineralization of native soil organic matter. In the present study, the rapid increase in soil pH under L3B may have relieved acid stress on soil microorganisms and enhanced microbial decomposition activity during the early stage, thereby contributing to the transient decline in SOM at 3–6 months. Similar mechanisms have been documented in alkaline-amended systems, where amendment-induced changes in substrate availability and soil chemical conditions can stimulate organic matter turnover [26]. The subsequent increase in SOM at 12 months suggests that the carbon-retention effect of biochar gradually became more evident as the soil system stabilized.

The differentiation of nitrogen forms provides important evidence for elucidating the yield-enhancement mechanism under the combined application. The combined application significantly increased TN and NO_3_^−^-N, whereas the sole application of biochar was more conducive to maintaining NH_4_^+^-N; this indicates that quicklime-driven acidity correction favors nitrification, while biochar favors NH_4_^+^-N retention [13,27]. Previous studies have shown that both quicklime and biochar can promote nitrogen transformation in acidic soils, although the former primarily enhances nitrification by elevating pH, while the latter more effectively improves the rhizosphere environment and increases nitrogen retention and utilization efficiency [13,27]. In the present study, yield was significantly positively correlated with pH, SOM, and NO_3_^−^-N, whereas the overall correlation with enzyme activities and microbial abundance was relatively weak, suggesting that under the experimental conditions yield formation was more directly governed by available nitrogen supply and improvements in the physicochemical environment. Therefore, the yield advantage of L2B did not arise from the maximization of any single indicator but from a better balance among pH elevation, SOM maintenance, and NO_3_^−^-N supply.

Phosphorus and potassium responses further reflect the synergistic regulation of multiple elements under the combined application. L1B and L2B exhibited more stable increases in TP, TK, and AK during the mid-to-late stages, indicating that the nutrient input and retention capacity of biochar can act synergistically with the mobilization effect of quicklime following alleviation of acidic constraints [14,19,28]. For potassium, its availability is primarily controlled by exogenous inputs, exchange adsorption, and leaching processes. Biochar can directly supply a certain amount of soluble potassium and, by increasing soil cation exchange capacity, enhance K^+^ retention and reduce leaching; therefore, it is more favorable for AK accumulation [28]. However, the highest AP values were still recorded under the sole application of biochar, and pH showed a significant negative correlation with AP, suggesting that stronger alkalization does not necessarily correspond to higher available phosphorus accumulation. The capacity of biochar to promote phosphorus availability may depend more on its direct phosphorus supply and the integrated regulation of adsorption and microbial processes [29,30]. Therefore, the fluctuations in ACP under the high-dose combined treatment (L3B) in the present study should be interpreted as a stage-dependent response to shifts in pH and substrate availability rather than solely as enhanced enzymatic mineralization. This indicates that phosphorus activation under combined application conditions is more likely driven jointly by pH regulation, transformations in phosphorus fractions, and microbial adaptation [29,30].

### 3.2. Combined Application of Biochar and Quicklime Improves Soil Biological Properties

Changes in enzyme activities and microbial abundances further support the above conclusions. L1B and L2B continuously increased UE and SC activities, indicating that moderate combined application simultaneously enhanced nitrogen transformation and labile carbon turnover [22,27]. However, L3B produced only limited improvement in UE activity, suggesting that stronger alkalization is not the optimal approach for maintaining soil biological activity [22,27]. Furthermore, the contrasting responses of UE and ACP under L3B suggest that stronger alkalisation may have altered microbial nutrient-acquisition strategies. The limited enhancement of UE under L3B, together with the pronounced fluctuation in ACP activity, may indicate a shift in microbial resource allocation from nitrogen acquisition to phosphorus acquisition under stronger alkalisation. This response reflects a potential stoichiometric adjustment, consistent with the framework of Liu et al. [31], in which soil nutrient balance and microbial nutrient demand jointly regulate nitrogen- and phosphorus-related processes. Both the combined application and the sole application of quicklime decreased ACP activity, indicating that ACP expression is more likely a demand-responsive type induced by acid stress and phosphorus limitation; when soil acidity is alleviated and P supply is improved, ACP secretion and activity tend to decline [30]. This also explains why the increases in TP and AP in the present study were not accompanied by a synchronous elevation in ACP activity. With regard to microbial abundance, bacteria and actinomycetes responded more strongly to the combined application, particularly under high-dose treatments, whereas fungal responses were less consistent and showed a negative correlation with pH and positive correlations with SOM, AP, and AK. This is consistent with recent findings that bacteria generally respond more directly to pH improvement, whereas fungal communities are more dependent on organic carbon supply [32,33,34].

The delayed increase in bacterial abundance under L1B and L2B may be attributed to the time required for microbial adaptation after amendment application. In the early stage, the rapid pH increase induced by quicklime may have caused a short-term pH disturbance or environmental filtering effect, which did not immediately translate into higher bacterial abundance [35]. In addition, although biochar can provide porous microhabitats and surface sites that promote microbial adhesion, proliferation, survival, and colonization, the formation of stable soil–biochar–microbe interfaces may require time under field conditions [36]. Therefore, the significant increases in bacterial abundance observed in L1B and L2B at 12 months may reflect a delayed but cumulative response to pH stabilization and gradual microbial colonization of biochar-derived microhabitats.

Taken together, the effects of combined application on soil biological properties may operate through two coexisting mechanistic pathways: pH elevation drives increases in bacterial and actinomycete abundances and promotes enzyme activity recovery, while SOM maintenance favors the preservation of fungal communities and certain enzyme activities. Nevertheless, the above interpretations remain mechanistic inferences at present and still require further support from metagenomic and functional gene evidence. Moreover, because the microbial data were obtained using culture-dependent plate counts, they should be interpreted as changes in culturable microbial counts rather than shifts in the total soil microbiota. Given the well-known great plate count anomaly, future studies should employ molecular approaches, such as 16S rRNA/ITS amplicon sequencing or metagenomics, to validate the effects of biochar and quicklime co-application on soil microbial communities [37,38].

### 3.3. Combined Application of Biochar and Quicklime Improves Fruit Yield and Quality

With respect to productive traits, the combined application did not synchronously improve all fruit indices but exhibited a clear functional differentiation. L1B was more favorable for improving single-fruit traits and kernel-related traits, whereas L2B was more advantageous for increasing per-plant yield. This indicates that improvements in some fruit traits do not necessarily translate synchronously into maximum per-plant yield [39,40]. Correlation analysis showed that yield was significantly positively correlated with pH, SOM, and NO_3_^−^-N, but exhibited relatively weak correlations with most enzyme activities and microbial abundances. These results suggest that, within the study year, optimization of the soil chemical environment had greater explanatory power for yield formation than soil biological indicators. This finding is consistent with conclusions from previous orchard ecosystem studies and global meta-analyses [10,34]. However, in perennial woody oil crops, the present study further demonstrates that the moderate-dose combined treatment (L2B) is more beneficial for balancing nutrient status and yield formation.

The combined application also significantly improved kernel nutritional quality and lipid composition. L2B increased soluble sugar and soluble protein contents, while L3B increased AA, VE, and oil contents. Meanwhile, both treatments shifted the fatty acid profile toward higher oleic acid and lower linoleic acid proportions. The observed shift toward higher oleic acid and lower linoleic acid contents under the combined treatments, especially L2B and L3B, may be partly associated with improved potassium and phosphorus availability. Previous studies have shown that K and P nutrition can influence oil accumulation and fatty acid composition in oilseed crops [41,42]. Therefore, the relatively high AK and AP levels maintained under the combined treatments during the mid-to-late stages may have contributed to the optimization of kernel fatty acid composition in pecan. Given the positive correlations of TN and TP with oleic acid and oil content, the present study suggests that improved nutrient supply not only influences yield formation but may also affect oil content and fatty acid composition by regulating kernel lipid synthesis and storage metabolism [41,42]. Previous studies have shown that oleic acid and linoleic acid are the predominant fatty acids in pecan kernels, with their ratio determining oxidative stability and nutritional value, and being jointly influenced by cultivar, harvest timing, and lipid biosynthesis regulation [43,44,45]. Since cultivar and harvest timing were consistent in the present study, the optimization of fatty acid composition is more likely associated with improvements in soil nutrient status and the rhizosphere environment. It should be emphasized that L3B outperformed L2B in terms of quality but did not further increase yield. This phenomenon indicates that stronger acidity correction does not necessarily translate into higher yield and may instead exert a greater influence on kernel filling and metabolic quality. Similar dose-dependent differentiation has also been reported in recent combined application studies [15].

## 4. Materials and Methods

### 4.1. Overview of the Experimental Site

The experimental site for this study was located at the pecan research base in Jingzhou Miao and Dong Autonomous County, Huaihua City, Hunan Province, China (109°28′ E, 26°23′ N; elevation 388 m). This region has a typical subtropical monsoon climate, characterized by warmth and humidity with distinct four seasons. The mean annual temperature is 16.8 °C, the mean annual frost-free period is 290 days, the mean annual precipitation is 1388.6 mm, and the mean annual sunshine duration is 1336.9 h. The soil type is Quaternary acidic red soil.

### 4.2. Experimental Materials and Design

The 8-year-old pecan cultivar ‘Jinhua’ was selected as the experimental material, with an average plant height of approximately 4.4 m, a crown width of approximately 5 m, and a planting spacing of 8 m × 8 m. The experimental plants exhibited vigorous growth, were free from pests and diseases, and displayed uniform tree architecture.

**Figure 8:**
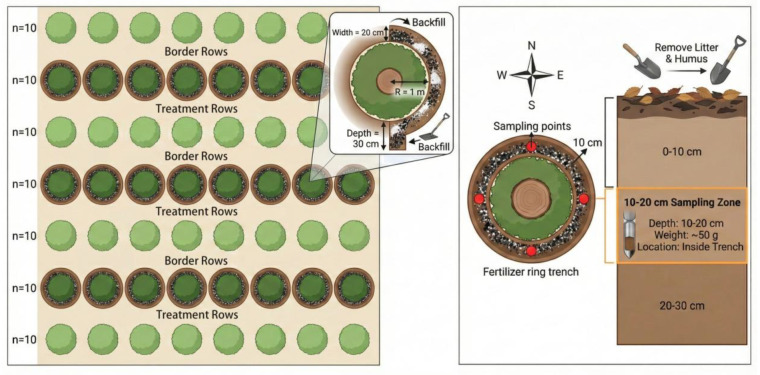
Field experiment layout map.

The field experiment was conducted over a 12-month period, from 26 December 2023, when the amendments were applied, to 26 December 2024, when the final soil sampling was completed. A total of eight treatments were established (Table 1). For each treatment, one row of sample trees (10 plants) was selected with 3 replicates; one row of trees was established as a guard row between treatments, resulting in a total of 240 pecan trees treated in this experiment (Figure 8). The test biochar was produced by burning corn straw, with the following basic physicochemical properties: pH 9.46, organic carbon content 42.21%, total nitrogen content 8.34%, total phosphorus content 2.31%, total potassium content 16.12%, and an application rate of 2 kg per plant. The biochar application rate was determined based on previous studies on biochar amendment in acidic soils and orchard systems [46,47,48]. The test lime was quicklime, and its application rates of 1–2 kg per plant were determined based on previous literature and a preliminary soil acidity neutralization titration test [49].

The amendments were applied on 26 December 2023, using annular trenching fertilization (Figure 1): an annular ditch was excavated 1 m from the trunk center, with a depth of 30 cm and a width of 20 cm, into which the biochar and quicklime were appropriately mixed with the soil before application, followed by backfilling with topsoil.

### 4.3. Sample Collection

Following treatment, sampling was performed once every 3 months for a total of 4 samplings, conducted on 26 March, 26 June, 26 September, and 26 December 2024, respectively. Soil sampling was conducted in the east, south, west, and north directions of each sample tree, with samples collected 10 cm inward from the edge of the fertilization trench. Prior to sampling, surface litter and the top humus layer were removed; approximately 50 g of soil was collected from the 10–20 cm tillage layer, placed in sealed bags with appropriate labeling, and promptly returned to the laboratory for subsequent soil property analyses.

After transporting the soil samples back to the laboratory, larger stones, roots, and plant residues were removed; the soil clods were crushed, sieved through a 2 mm mesh, and thoroughly homogenized. The sieved soil samples were divided into three portions: (1) one portion of fresh soil was stored at −20 °C for the determination of ammonium nitrogen (NH_4_^+^-N) and nitrate nitrogen (NO_3_^−^-N); (2) one portion of fresh soil was stored at 4 °C for the determination of soil microbial populations and enzyme activities; (3) the remaining soil was air-dried, with the air-dried soil passed through a 1 mm sieve for the measurement of soil pH, total potassium (TK), available phosphorus (AP), and available potassium (AK), and through a 0.149 mm sieve for the determination of soil organic matter (SOM), total nitrogen (TN), and total phosphorus (TP).

Mature fruit samples were collected on 15 October 2024: all fruits from the sample trees under each treatment were harvested for determination of fresh fruit yield (FY); simultaneously, 10 fruits were collected from each of the east, south, west, and north directions per sample tree for the assessment of fruit physical characteristics and internal constituents.

### 4.4. Determination of Soil Samples

The treatment and determination methods for routine physicochemical properties of soil followed the internationally accepted soil chemical analysis system [50]. Soil pH was determined using a Lei Ci PHS-3C acidity meter (LEICI, PHS-3C, Shanghai, China) after shaking at a soil to sterile water ratio of 1:2.5 for 30 min and standing for 2 h; SOM was determined by the potassium dichromate external heating method [51]; TN was determined using a fully automatic chemical analyzer (SmartChem 200, West Co Scientific Instruments, Rome, Italy) [52]; TP was determined by the molybdenum–antimony colorimetric method following NaOH fusion treatment; TK was determined by flame photometry following NaOH fusion treatment; AP was determined by the molybdenum–antimony colorimetric method after joint extraction with hydrochloric acid and ammonium fluoride; AK was determined by flame photometry after extraction with ammonium acetate (NH_4_OAc); NH_4_^+^-N and NO_3_^−^-N were determined using a fully automatic discrete analyzer (SmartChem200, West Co Scientific Instruments, Rome, Italy) [53].

Soil enzyme activities were determined according to classical soil enzymology methods: urease (UE) activity was measured by colorimetry [54]; sucrase activity (SC) was determined according to the soil invertase procedure by the DNS method for reducing sugar color development [55,56]; acid phosphatase (ACP) activity was determined by the p-nitrophenyl phosphate (pNPP) substrate colorimetric method [57,58]; catalase activity (CAT) was determined by the potassium permanganate titration method [59]. The populations of soil bacteria (TPC), fungi (TFC), and actinomycetes (TAC) were determined by the dilution plate counting method, and the results were expressed as CFU·g^−1^ soil [60,61,62].

### 4.5. Determination of Fruit Samples

At the fruit maturity stage, all fruits from the sample trees were fully harvested according to treatment, and the average value was calculated to obtain the fresh fruit yield per tree for different treatments. The fresh fruit weight (FFY), fresh seed weight (FSW), dry seed weight (DSW), and dry kernel weight (DKW) of pecan were determined using a 0.001 g analytical balance; the fruit transverse diameter (FTD) and vertical diameter (FVD) were measured using a vernier caliper with a precision of 0.001 mm; the kernel oil content was determined by Soxhlet extraction [63]. The fruit shape index (FSI), fresh seed yield (FSY) (%), dry seed yield (DSY) (%), and dry kernel yield from dry seed yield (DKY) (%) were calculated according to the following formulas:

FSI = FVD (mm)/FTD (mm)(1)

FSY (%) = FSW (g)/FFY(g) × 100%(2)

DSY (%) = DSW (g)/FSW(g) × 100%(3)

DKY (%) = DKW (g)/DSW(g) × 100%(4)

The soluble sugar content of the fruit (SS) was determined by the anthrone–sulfuric acid colorimetric method, in which soluble sugars extracted from fruit tissue react with anthrone under acidic conditions to form a colored complex for spectrophotometric quantification [64]. The soluble protein (SP) content was determined by the Coomassie brilliant blue method, based on the binding of the dye to proteins and the resulting absorbance change [65]. The amino acid content (AA) was determined by the ninhydrin colorimetric method, in which free amino acids react with ninhydrin to produce a colored compound for quantitative analysis [66]. Vitamin E (VE) was determined by high-performance liquid chromatography, in which tocopherol compounds were extracted, chromatographically separated, and quantified against standards [67,68]. The fatty acid composition was quantitatively determined by gas chromatography after preparation of fatty acid methyl esters according to the internal standard method [69].

### 4.6. Data Statistics and Analysis

Data were recorded and preliminarily organized using Excel 2021. Means and standard deviations were calculated, and statistical analyses were performed using SPSS 26.0. One-way analysis of variance was conducted using SPSS 26.0 to examine the significant differences in soil fertility factors and fruit economic traits among the different treatments. Pearson correlation analysis was employed to evaluate the relationships between soil chemical properties, biological properties, and fruit yield and quality. For statistical analysis, each replicate plot, rather than each individual tree, was used as the experimental unit. Measurements from individual trees within each replicate plot were averaged before analysis, and the three replicate plot means for each treatment were used for one-way ANOVA and Pearson correlation analysis. Figures were prepared using Origin 2025 software.

## 5. Conclusions

The combined application of biochar and quicklime effectively alleviated soil acidity and improved soil fertility, soil biological properties, fruit yield, and kernel quality in acidic red soil pecan orchards. Compared with the control, the combined treatments increased soil pH by 0.47–2.15 units and improved soil nutrient availability; at 12 months after application, SOM under L1B, L2B, and L3B increased by 36.31%, 31.86%, and 18.08%, respectively, while TN was 126.0–166.7% higher than that under the corresponding quicklime-only treatments. The combined treatments also maintained relatively high levels of TP and AP and enhanced soil biological indicators, particularly bacterial and actinomycete abundances at the later sampling stages. The effects of the combined treatments differed according to the biochar–quicklime ratio: L1B (2 kg biochar + 1 kg quicklime per plant) was more effective in improving fruit physical traits, including fresh fruit weight, fresh seed weight, dry seed weight, dry kernel weight, and dry kernel yield; L2B (2 kg biochar + 1.5 kg quicklime per plant) showed the greatest yield-enhancing effect, with single-tree yield reaching 26.80 kg, 24.25% higher than that of the control; and L3B (2 kg biochar + 2 kg quicklime per plant) was more favorable for improving kernel nutritional quality, increasing amino acid and VE contents by 97.09% and 170.80%, respectively, and increasing oil content to 57.43%. In addition, the combined treatments improved fatty acid composition by increasing oleic acid content and reducing linoleic acid content, with L2B and L3B showing the most pronounced effects. For practical orchard management under conditions similar to those of this study, we recommend applying 2 kg biochar plus 1.5 kg quicklime per plant during the dormant season using annular trenching approximately 1 m from the trunk, 30 cm deep, and 20 cm wide, because this treatment provided the best balance between soil fertility improvement and yield enhancement. Where kernel nutritional quality is prioritized over maximum yield, 2 kg biochar plus 2 kg quicklime per plant may be considered. Future studies should further verify these recommendations through multi-year field monitoring, integrated analyses of soil microbial and nutrient-cycling mechanisms, and comprehensive economic evaluations.

## Figures and Tables

**Table 1:** The application rates of biochar and quicklime in each experimental treatment.

Treatment	Biochar (kg/Plant)	Quicklime (kg/Plant)
CK	0.0	0.0
B	2.0	0.0
L1	0.0	1.0
L2	0.0	1.5
L3	0.0	2.0
L1B	2.0	1.0
L2B	2.0	1.5
L3B	2.0	2.0

## Data Availability

The data presented in this study are available on reasonable request from the corresponding author. The data are not publicly available at this stage because they are part of an ongoing research project and are subject to institutional data management requirements.
